# HCC surveillance results in earlier HCC detection: results from an Indian cohort

**DOI:** 10.1186/2193-1801-3-610

**Published:** 2014-10-17

**Authors:** Anita Kohli, Allison A Murphy, Chirdeep Agarwal, Bhavana Shivakumar, Shyam Kottilil, Michael A Polis, G Mani Subramanian, Vandana Midha, Omesh Goyal, Srinivas Desai, Ajit Sood, Samir Shah

**Affiliations:** Laboratory of Immunoregulation, National Institute of Allergy and Infectious Diseases, National Institutes of Health, Bethesda, MD USA; Department of Gastroenterology, Jaslok Hospital, Mumbai, India; Dayanand Medical College & Hospital, Ludhiana, India; Department of Radiology, Jaslok Hospital, Mumbai, India; Clinical Research Directorate/Clinical Monitoring Research Program, Leidos Biomedical Research, Inc. (formerly SAIC-Frederick, Inc.), Frederick National Laboratory for Cancer Research, Frederick, MD 21702 USA; Gilead Sciences, Foster City, CA USA

**Keywords:** Hepatocellular carcinoma, Surveillance, Ultrasounds, Survival, Treatment, India

## Abstract

Hepatocellular carcinoma (HCC) is the third most common cause of cancer-related death worldwide, with an increased incidence in South Asia. In order to describe the effect of surveillance for HCC with biannual ultrasound and alpha-fetoprotein (AFP) on diagnosis and survival in an Indian population a retrospective cohort-control study was performed at two liver clinics in India. The medical records of 3,258 patients with cirrhosis who received surveillance for HCC were reviewed, and 100 patients who developed HCC identified. Sixty-four cirrhotic patients diagnosed with HCC during the same time period without a history of surveillance were included and survival, BCLC stage at diagnosis, and treatment were compared.

Patients who underwent surveillance were more likely to be diagnosed with potentially curable or treatable BCLC Stage 0/A disease and Stage B/C disease respectively, than late Stage D disease (χ2 = 0.0007). Patients diagnosed at an earlier stage of HCC lived significantly longer after diagnosis than patients diagnosed at a later stage (Stage 0/A: 15.6 ± 14.2 months vs. Stage B/C: 9.43 ± 19.7 months vs. Stage D: 5.59 ± 11.9 months; p = 0.0006). While treatment for HCC improved overall survival, only 28% of eligible patients received treatment, explaining the lack of survival benefit noted in the surveillance group. Surveillance for HCC led to detection of HCC at earlier stages. The impact of surveillance on improved mortality could not be evaluated given the limited number of patients who received treatment. HCC surveillance has the potential to improve survival in South Asian patients with cirrhosis only if improvements in access to appropriate treatment are made.

## Introduction

Hepatocellular carcinoma (HCC) is the third most common cause of cancer-related death worldwide, with an increased incidence in South East Asia. In India there are 19,000 new cases of HCC and 17,000 deaths due to HCC annually (Ferlay et al. 2010). Given the prevalence of two major risk factors for HCC in India, chronic hepatitis C (HCV) (1–1.9% population) (Sievert et al. 2011) and active hepatitis B (HBV) (~2.4%) (Batham et al. 2007), in combination with an aging population of patients expected to develop advanced liver disease, the incidence of HCC in India can be expected to increase.

Overall, prognosis after diagnosis of HCC is dependent on the stage of disease as well as the availability of appropriate individualized treatment modalities (Forner et al. 2010). In this regard, strategies for detection HCC at an early stage have been evaluated. Surveillance of high-risk patients with hepatitis and/or cirrhosis using biannual ultrasound and serum AFP testing has been shown to enable earlier detection of malignant tumor and prolong survival (Zhang et al. 2004) in a Chinese population, as well as to be a cost effective strategy in a US population (Bruix and Sherman 2011).

Limited retrospective studies on the epidemiology of HCC in India have been performed, most of which report that patients initial presentation is at an advanced stage of HCC disease (Kumar et al. 2008). In this study, we investigated the impact of existing standard HCC surveillance procedures on survival in two distinct geographic regions in India.

## Methods

A retrospective cohort-control study was performed at two specialty liver clinics in Mumbai, Maharashtra (western India) and Ludhiana, Punjab (northern India). The medical records of 3,258 patients with cirrhosis due to HBV, HCV or alcohol use who received surveillance for HCC for a minimum of 12 months were reviewed. Surveillance consisted of an abdominal ultrasound examination and serum alpha-fetoprotein testing every 6 months for at least 12 months. All ultrasounds were performed by trained radiologists. One hundred patients who developed HCC between July 1999 and Aug 2012 were identified for inclusion. An additional 64 cirrhotic patients from Mumbai without a history of surveillance, who at first presentation were diagnosed with HCC during the same time period, were included for comparison. Patients who received treatment for HCC were, in most cases, treated with therapeutic strategies consistent with the Barcelona Clinic Liver Cancer (BCLC) system (Bruix and Llovett 2002). Combination therapy was used when clinically indicated. For patients who received sequential therapy, the first therapy received is reported. Baseline demographics including risk factors for HCC (history of alcohol abuse (ETOH), HBV status and HCV status), BCLC stage at diagnosis, treatment received, and survival were recorded using a standardized questionnaire.

Informed consent was obtained from patients where appropriate. The IRB of the National Institutes of Allergy and Infectious Disease, NIH, Bethesda, MD approved this study and all investigations have been carried out in accordance with the principles of the Declaration of Helsinki as revised in 2000.

### Statistical analysis

Statistical analyses were performed using SAS Version 9.2 and Prism version 5.0. Categorical values were compared using the Pearson Chi-square test. Two-tailed Student’s T-tests were used to compare means. A p-value of <0.05 was considered significant. Kaplan-Meier curves were used to compare survival rates between groups.

## Results

### Demographics

Patient demographics are shown in Table 1. The majority of patients were men (83.5%). There was no significant difference in the age, gender, stage of liver disease at HCC diagnosis or risk factors for HCC between patients who received surveillance as compared to those who did not receive surveillance. Patients who underwent surveillance were more likely to present with Child-Pugh C disease than patients who did not receive surveillance. AFP was elevated (>20 ng/mL) in 76.8% of patients at the time of HCC diagnosis, with no difference between patients who did or did not undergo surveillance (p = 0.15).

**Table 1:** **Demographics of patients diagnosed with HCC who did and did not undergo surveillance**

	Surveillance (n = 100)	No surveillance(n = 64)	
**Age**- mean ± SD (years)	58 ± 9.6	59 ± 10.5	p = 0.70
**AFP**- mean ± SD (ng/mL)	2424 ± 9475	10275 ± 63429	p = 0.33
**Size of lesions**- mean ± SD (cm)	5.00 ± 2.71	5.73 ± 3.42	p = 0.23
**Gender**- male n (%)	85 (85%)	52 (81%)	χ^2=^0.53
**Child-pugh class**			
A	22 (22%)	10 (16%)	χ^2=^0.04
B	36 (36%)	36 (56%)	
C	42 (42%)	18 (28%)	
**Etiology**			
**HBV**			χ^2=^0.42
HBV only	7 (7%)	3 (5%)	
HBV & ETOH	1 (1%)	0 (0%)	
**HCV**			
HCV only	23 (23%)	19 (30%)	
HCV & ETOH	17 (17%)	14 (22%)	
**ETOH only**	26 (26%)	9 (14%)	
**HBV & HCV & ETOH**	23 (23%)	17 (27%)	
**Unknown**	3 (3%)	2 (3%)	

### Surveillance result in earlier detection of HCC

Patients with cirrhosis who underwent HCC surveillance were more likely to be diagnosed with HCC that was either potentially curable (BCLC Stage 0/A) or with HCC eligible for treatment with life-prolonging therapies (Stage B/C disease) than patients who did not undergo surveillance (Stage 0/A: 11% surveillance vs. 7% no surveillance; Stage B/C 66% surveillance vs. 42% no surveillance; Stage D 23% surveillance vs. 51% no surveillance vs.; χ^2^ = 0.0007) (Table 2).

**Table 2:** **Patients undergoing surveillance diagnosed with HCC at earlier stages**

	Surveillance(n = 100)	No surveillance (n = 64)	
**BCLC stage at diagnosis**			
0/A	11 (11%)	4 (7%)	χ^2=^0.0007
B/C	66 (66%)	27 (42%)	
D	23 (23%)	33 (51%)	

### Surveillance improves overall survival in patients with access to appropriate HCC treatment

**Figure 1:**
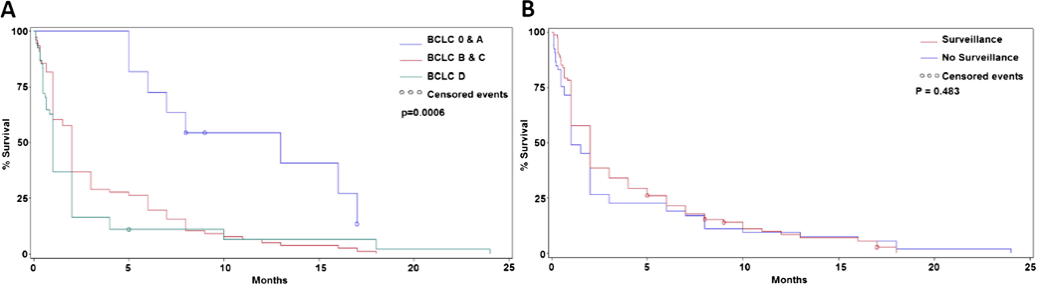
**Impact of disease stage and surveillance on survival. A**. Improved Survival After Early Diagnosis of HCC. Patients diagnosed at an earlier stage of HCC lived significantly longer after diagnosis than patients diagnosed at a later stage (Stage 0/A: 15.6 ± 14.2 months vs. Stage B/C: 9.43 ± 19.7 months vs. Stage D: 5.59 ± 11.9 months; p = 0.0006). **B**. Surveillance for HCC Does Not Improve Survival. Despite the finding that surveillance led to increased diagnosis of early stage HCC, we did not find a difference in the overall survival of patients who did and did not undergo routine surveillance (p > 0.05).

Patients diagnosed at an earlier stage of HCC lived significantly longer after diagnosis than patients diagnosed at a later stage (Stage 0/A: 15.6 ± 14.2 months vs. Stage B/C: 9.43 ± 19.7 months vs. Stage D: 5.59 ± 11.9 months; p = 0.0006) (Figure 1A). Despite the finding that surveillance led to diagnosis of HCC at earlier stages, we did not find a difference in the overall survival of patients who did and did not undergo routine surveillance (p = 0.48) (Figure 1B).

**Figure 2:**
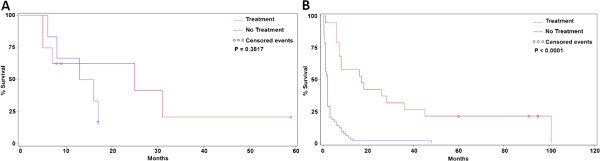
**Impact of Treatment for HCC on Survival. A.** Survival in Treated Patients with BCLC Stage 0/A Hepatocellular Carcinoma. Survival in the small number of patients with Stage 0/A HCC was similar between treated and untreated patients (20.3 ± 25.9 months vs. 11.0 ± 5.4 months for untreated patients; p = 0.3817). **B**. Treatment Improves Survival Patients with BCLC Stage B/C Hepatocellular Carcinoma. Patients with Stage B or C disease survived longer as compared patients of the same stage who did not receive treatment (33.0 ± 34.9 months treated vs. 4.8 ± 8.2 months untreated; p < 0.0001).

Overall, only 28% of patients with BCLC stage 0-C disease at the time of diagnosis received treatment for HCC treatment. Patients with stage 0/A disease were more likely to receive treatment than patients diagnosed with stage B/C or stage D HCC (53.3% of Stage 0/A disease treated, 22.0% of Stage B/C disease treated and 10.2% of Stage D treated, χ^2^ = 0.0016). Among patients who were treated, the most common treatment modalities are shown in Table 3. There was no difference in treatment modalities used in patients who did or did not undergo surveillance (χ^2^ < 0.05).Patients who received potentially curative treatment for Stage 0 or A disease (Figure 2A) or life-prolonging therapy for Stage B or C disease (Figure 2B) survived longer as compared patients of the same stage who did not receive treatment (Stage 0/A: 20.3 ± 25.9 months treated vs. 11.0 ± 5.4 months untreated, p = 0.3817; Stage B/C: 33.0 ± 34.9 months treated vs. 4.8 ± 8.2 months untreated, p < 0.0001). However, this difference was significant only for patients with Stage B/C disease. Using a logistic regression model including age, gender and stage of HCC and Child-Pugh score, only Child-Pugh Scores at diagnosis was a risk factor for not receiving treatment (p = 0.0005).

**Table 3:** **Treatment of Hepatocellular Carcinoma in India**

BCLC stage	No treatment n (%)	Chemo-embolization n (%)	Resection n (%)	Sorafenib n (%)	Liver transplant n (%)	ETOH injection n (%)	RFA n (%)	TACE n (%)
**0**	1 (5.00%)	0	0	0	0	0	1 (50.0%)	0
**A**	6 (46.0%)	3 (21.4%)	1 (7.7%)	0	1 (7.7%)	0	2 (14.3%)	0
**B**	36 (69.2%)	5 (9.6%)	5 (9.6%)	2 (3.9%)	0	0	2 (3.9%)	2 (3.9%)
**C**	29 (87.9%)	1 (3.0%)	0	2 (6.1%)	0	1 (3.0%)	0	0
**D**	53 (88.3%)	1 (1.7%)	0	0	3 (5.0%)	0	0	2 (3.3%)

*RFA* Radiofrequency Frequency Ablation.

*TACE* Transarterial Chemoembolization.

## Discussion

In this study, we demonstrate that systematic surveillance for HCC in cirrhotic patients leads to earlier diagnosis of HCC in India. In addition early diagnosis and appropriate therapy for HCC did prolong survival. We did not find that surveillance improved overall survival, however treatment for HCC after diagnosis was uncommon in this cohort limiting the ability to make conclusions about the impact of surveillance on survival. Findings in this study support the efficacy of biannual U/S and AFP testing as an HCC surveillance strategy to detect early stage disease in a South Asian population. Efforts to understand barriers to the receipt of treatment for HCC will be important in order to improve mortality in Indian patients with HCC.

HCC is a large and increasing problem in India given the high prevalence of risk factors, including viral hepatitis, in the general population. Infection with viral hepatitis (HBV and/or HCV) as a risk factor for HCC in our cohorts reflected the distribution of viral hepatitis in India, with about half of the cases related to HBV and half of the cases related to HCV. Men were predominantly affected by HCC, again, reflecting the epidemiology of risk factors for cirrhosis (El-Serag 2012). The majority of patients in this study had a history of excessive alcohol use and one quarter of patients had alcohol as their only risk factor for HCC, suggesting barriers to further medical treatment in this traditionally difficult-to-retain patient population. Diagnostic level of serum AFP was seen in many but not all patients at the time of HCC diagnosis, similar to other reports describing the presentation of HCC in India (Kumar et al. 2008), and highlighting the limited utility of this assay alone as a screening tool (Oka et al. 1994).

Given the poor prognosis and insidious onset of late stage HCC, efforts to develop effective surveillance measures to detect disease at an early stage in high-risk patients, when curative or life-prolonging therapies are more effective, are ongoing. Biannual screening using ultrasound with or without AFP as done in this study is the gold standard for the screening of HCC with a sensitivity of 57-100% (Snowberger et al. 2007; Singal et al. 2012). A single randomized controlled trial showed a 37% reduction in mortality despite a relatively low compliance rate with screening of 60% (Zhang et al. 2004). Overall however, these data reiterate the need for novel biomarkers that can detect HCC at earlier time point than present surveillance strategies, which require frequent visits and have variable sensitivity.

In our cohort, most patients presented with an advanced stage of HCC, similar to other Indian cohorts (Kumar et al. 2008). We, however, were able to detect stage-migration, which is the ability of biannual ultrasound and AFP surveillance to detect disease at an earlier stage as compared to patients who did not undergo surveillance. Surveillance tools however, are effective at reducing mortality only when diagnosed patients are linked to effective, life-prolonging treatments (Di Bisceglie 2004). We did not find a direct effect of surveillance on mortality reduction. This is likely due to the low proportion of patients who received treatment for HCC. Survival however, was prolonged for treated patients compared to those who did not receive treatment, with success rates comparable to those reported in other studies (de Lope et al. 2012; Cillo et al. 2006). This suggests that routine surveillance to diagnose patients with early curable or treatable disease coupled with linkage to appropriate therapy could improve mortality due to HCC in this Indian population.

Access to appropriate treatment HCC in India is frequently impeded by barriers. In particular, living donor or cadaveric liver transplantation, an important and potentially curative therapeutic strategy, is rare in India, with only two active transplant centers performing about 1,000 transplants per year. Larger studies to determine the efficacy of current screening strategies as well as to understand and mitigate barriers to care for patients in India (cost, access to transplant centers, alcohol use) with HCC are critical.

Limitations of this study include retrospective design, retrospective classification of HCC disease and BCLC staging criteria and small numbers of treated patients with HCC, thereby restricting the ability of this study to detect survival advantages in the surveillance or non-surveillance groups.

Our study emphasizes three important points. First, vigilant surveillance strategies for HCC in patients who have cirrhosis is vital for early detection. Second, in order to improve the extremely low overall survival rates observed even in the surveillance group, better access/linkage to care with early individualized therapeutic modalities are absolutely necessary to have an impact on the overall morbidity and mortality associated with HCC in India.
